# Using inhaled corticosteroids can reduce the decline of lung function in asthmatics: pilot study

**DOI:** 10.1186/1939-4551-8-S1-A200

**Published:** 2015-04-08

**Authors:** Paula Almeida, Adelmir Souza-Machado, Eduardo Ponte, Alvaro Cruz

**Affiliations:** 1 Universidade Federal DA Bahia, Brazil; 2 Proar-Núcleo De Excelência Em Asma Da Ufba, Brazil; 3 Proar - Faculdade De Medicina Da Bahia, Ufba, Brazil

## Background

Patients with asthma may accelerate the average decline in lung function that is related to ageing (±30ml/year) [1]. The aim of this study was to identify risk factors for decline in lung function in patients with severe asthma.

## Methods

Prospective cohort with 9 years of follow up. Inclusion criteria: diagnosis of asthma and at least one year of use of inhaled corticosteroids before enrollment. Every three months patients had a multidisciplinary evaluation and they received free medication (inhaled corticosteroids, long-acting and short-acting β-agonists) monthly. Spirometry was performed anually in a Koko® spirometer according to the ATS protocol. Brazilian standards reported by Pereira [2] were used as normal reference values.

## Results

Interim analysis of 94 patients is present herein. Eighty(85.1%) patients were female. Their mean age(+SD) was 53.37(+14.19)years. 49(52.1%) of them were overweighted or obese according to bioimpedance measurements. Eigth patients(8.51%) had no immediate reversibility after bronchodilator use, measured by FEV_1_. Median (interquartile range)values of lung function at baseline were: FEV_1pre-BD_1.76L(1.38-2.2) - 67%(55.25-81.5) of predicted, FEV_1post-BD_1,86L(1.55-2.41)- 75%(63-88). After 9 years of follow up, lung function values were: FEV_1pre-BD_1.54L(1.21-1.97)-64.3%(50.6-76.3), FEV_1post-BD_1.77L(1.38-2.23)-71.04%(57.64-83.43).Reversibility to bronchodilator was stable throughout the study. During the follow up,there was a decline of 10mL/year in FEV_1(post-BD)_. Thirty patients had uncontrolled asthma by ACQ score in their last evaluation. Among these patients, we found lower lung function and more reversibility to bronchodilator in the end of the study with FEV_1pre-BD_1,35L(1,15-1,81), indicating a reduction of 45mL/year in basal function, but they have better reversibility with median(IR) 275mL(153-388) and 17.3%(11.16-29.6). 56.4% patients had positive skin prick test to aeroallergens. Lung function in patients with a negative test, was lower [median (IR) FEV_1pre-BD_1.42L(1.14-1.81)] in comparison to FEV_1pre-BD_1.67L(1.26-2.23)among atopics, the non atopics presenting a decline of 38mL/year in their lung function, not significantly different from the atopics. Only 9 patients did not have rhinitis. They had the lowest lung function with a FEV_1pre-BD_1.22(1.10-1.38), with a loss of 60mL/year in FEV_1pre-BD_.After 9 years of regular treatment ACQ score improved significantly [median points (IR) 0.83(0.33-1.83)].

## Conclusions

Long term treatment controls symptoms of asthma and protects against decline in lung function. Uncontrolled asthma, nonatopic asthma and the absence of rhinitis appear to be related to a trend to a increased decline, though not statistically significant.
